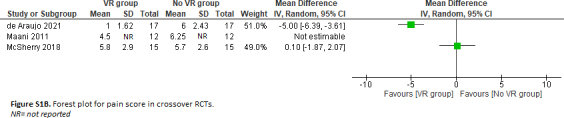# The effect of virtual reality therapy on pain and anxiety during wound care in adults: A systematic review

**DOI:** 10.1016/j.heliyon.2024.e40858

**Published:** 2024-12-07

**Authors:** H. Demirci, T. Lachkar, W.X.I. Fleur, E.Z. Barsom, A.M. Eskes, M.P. Schijven

**Affiliations:** a Amsterdam UMC Location University of Amsterdam, Surgery, De Boelelaan 1117, Amsterdam, the Netherlands; b Amsterdam UMC Location University of Amsterdam, Surgery, Meibergdreef 9, Amsterdam, the Netherlands; c Amsterdam Gastroenterology and Metabolism, Amsterdam, the Netherlands; d Amsterdam Public Health, Digital Health, Amsterdam, the Netherlands; e School of Nursing and Midwifery, Griffith University, Gold Coast, G01 2.03, Gold Coast Campus Griffith University, QLD, 4222, Australia

## 1 Introduction

Yearly, 310 million patients are estimated to undergo major surgery [1]. Most wounds are closed by *primary closure* at the end of surgery, but some wounds are deliberately left open or reopened by the surgeon if they are highly contaminated or at risk of infection [2]. Wound dehiscence, a postoperative complication, occurs when wounds are closed under stress, in ischemic tissue or if infection occurs, leading to reopening [3]. Consequently, approximately 28 % of surgical wounds undergo *secondary healing*, also known as *healing by secondary intention*, where the wound is left open to heal naturally [2,4]. In such cases, wound treatment can cause pain due to stress on the tissue and activation of sensory neurons combined with mental stress, which can lead to suboptimal care and impeded healing [[5], [6], [7], [8], [9], [10], [11], [12]].

Chronic wounds are a major health care problem, with significant clinical and social consequences [4]. The annual cost of wound care in the U.S. exceeds $20 billion and is increasing 10 % annually [4]. Supporting wound care with new technologies that improve adherence and relieve pain could significantly reduce costs for patients [13].

Various interventions are used to prevent or mitigate pain during wound care, e.g. the use of local or topical anaesthetics, oral pain medication, anxiolytics, and more patient-friendly (non-adhesive) dressings [5,10,14]. The use of Virtual Reality (VR) seems to be a promising tool in this perspective, having the potential to mentally distract patients from experiencing pain [15]. In recent years, VR technology development has increased. Both software and hardware have improved significantly and are evolving to date [[16], [17], [18]]. In the medical domain, VR is increasingly used with promising effects on lowering pain and anxiety in children [19,20]. There is also evidence that VR can reduce acute and chronic pain during procedural interventions in adults. But to date, there is rather limited evidence on the effects of VR in reducing pain during wound care [21]. Hence, it remains unclear if VR is indeed an effective intervention to reduce pain, and anxiety during wound care in adults. The systematic review by Dreesman et al. described the effect of VR therapy on pain and anxiety during wound care in adults in 2020, but also included studies investigating the effect of VR in setting other than wound care [22]. This updated systematic review aims to assess the impact of VR on pain and anxiety during wound care in adults, both in hospital and outpatient settings.

## 2 Methods

The systematic review and meta-analysis of this research is reported according to the Preferred Reporting Items for Systematic Review and Meta-Analyses (PRISMA) statement and performed according to the guidelines as reflected in the Cochrane Handbook for systematic reviews of interventions [23,24]. This study was prospectively registered in the PROSPERO database (registration No.CRD42022338137).

### 2.1 Eligibility criteria

Studies that investigated the effectiveness of Virtual Reality Therapy compared to provision of ‘care as usual’, e.g. provision of wound care that is not supported by VR or by any other distracting intervention in adults (≥18 years), regardless of the setting (in-or outpatient) and type of wound. Studies were considered eligible when inclusion criteria were met and the study reported on one or more of the predefined outcome parameters. The primary outcome parameter was pain score. Secondary outcome parameters included anxiety, use of pain medication, patient satisfaction, systolic blood pressure (SBP), diastolic blood pressure (DBP), blood oxygen levels (SaO_2_), heart rate (HR), and respiration rate (RR) [25]. In addition, provider satisfaction responses were collected, measured with questionnaires where rating scales were used. Excluded from search were trial protocols, conference abstracts and proceedings, secondary publications of previously published studies, commentaries and articles without available full text, reviews, letters, abstracts, comments, editorial, case reports, and case series. Publications in languages other than English were excluded as well.

### 2.2 Database search and data collection

The online databases of PubMed, Cochrane Library, CINAHL (through Ebsco), and Embase (through Ovid) were searched for articles published up to May 28, 2024. The following Mesh terms were used in the search in all conceivable combinations, using all available synonyms: ‘’Virtual Reality’, ‘’Virtual Reality Exposure Therapy’’, ‘’Augmented Reality’’, ‘’Video’’, ‘’Mixed Reality’’, ‘’Wounds and Injuries'’, ‘’Pain’’, ‘’Pain management’’, ‘’Anxiety’’, ‘’’Fear’’, and ‘’Bandages'’. The literature search was performed together with the clinical librarian (F.S.). Duplicates were removed. See Appendix 1 for the complete search strategy.

*Study selection* Two reviewers independently screened all titles and abstracts for eligibility criteria (HD and TL) and performed the full text screening in Rayyan [26]. A third reviewer (AE or MS) was consulted to resolve disagreements.

### 2.3 Data extraction

Two reviewers (HD and WF) independently extracted the data from the included studies. The following data were extracted using a predefined form: author, publication year, country, age, sex, and number of patients, duration of study, type-of wound, wound care, type- and duration of intervention, pain scores before- and after wound care procedure; anxiety score before and after; pain medication use; patient- and provider satisfaction; vital signs, such as SBP, DBP, HR, SaO_2_, and RR. Corresponding authors were contacted by e-mail if data were unclear, incomplete or if studies included both children and adults and data from adult patients was not described separately. Disagreements were resolved by contacting a third reviewer (AE or MS).

### 2.4 Methodologic quality assessment

The quality of the included randomized controlled trials (RCTs) and crossover RCTs were assessed independently by two reviewers (HD and WF), according to the Cochrane Collaboration tool for assessing the risk of bias [27]. For RCTs, the following items were evaluated: randomisation process, deviations from the intended interventions, missing outcome data, measurement of the outcome, selection of the reported results, and overall risk. For cross-over RCTs the items were slightly different and included: randomisation process, bias arising from period and carryover effects, deviations from the intended interventions, missing outcome data, measurement of the outcome, selection of the reported result, and overall risk. All items were scored as low, high, or unclear risk of bias. In case of disagreement, the third reviewer (AE or MS) was contacted to resolve [28].

### 2.5 Data synthesis

The primary outcome parameter was pain score, as measured using either the Visual Analog Scale (VAS), Graphic Rating Scale (GRS), Verbal Numeric Scale (VNS) or Numeric Rating Scale (NRS). All scales referred to a 10-cm continuous scale with endpoint '0′ representing ‘no pain' and endpoint '10’ representing ‘intolerable pain'. Mean difference (MD) and 95 % confidence interval (CI) were calculated. If studies only reported medians and interquartile ranges, the method proposed by Wan et al. was used to calculate the mean and standard deviation (SD) enabling data comparison in a meta-analysis [28,29]. Statistical heterogeneity among studies was evaluated using the I^2^ statistic where values exceeding 75 % were considered as substantial heterogeneity [30,31]. A *p*-value <0.05 was considered statistically significant. RevMan5.4 statistical software was used to conduct the meta-analysis [32].

## 3 Results

### 3.1 Search results

A total of 2662 articles were obtained through the literature search till May 28, 2024. After removal of duplicates (525), 2137 articles were screened for eligibility. Of these articles, 2099 articles did not meet the inclusion criteria, hence were excluded. As well as 4 studies which reported outcomes for both children and adults, where adult outcomes could not be separated. Four authors were contacted to obtain results from adult patients only. Two authors had no separate results from the adult patients [33,34] The authors of the other two studies did not respond, therefore these articles were excluded [35,36]. One author was contacted to obtain additional information about the unclear data, who did not respond [37]. One additional article was found eligible through cross-referencing. After full-text screening of 35 articles, 25 more articles were excluded. In total, 10 articles were included in qualitative synthesis [28,[37], [38], [39], [40], [41], [42], [43], [44], [45]]. Fig. 1 shows the flow chart of the selection process.

**Fig. 1:**
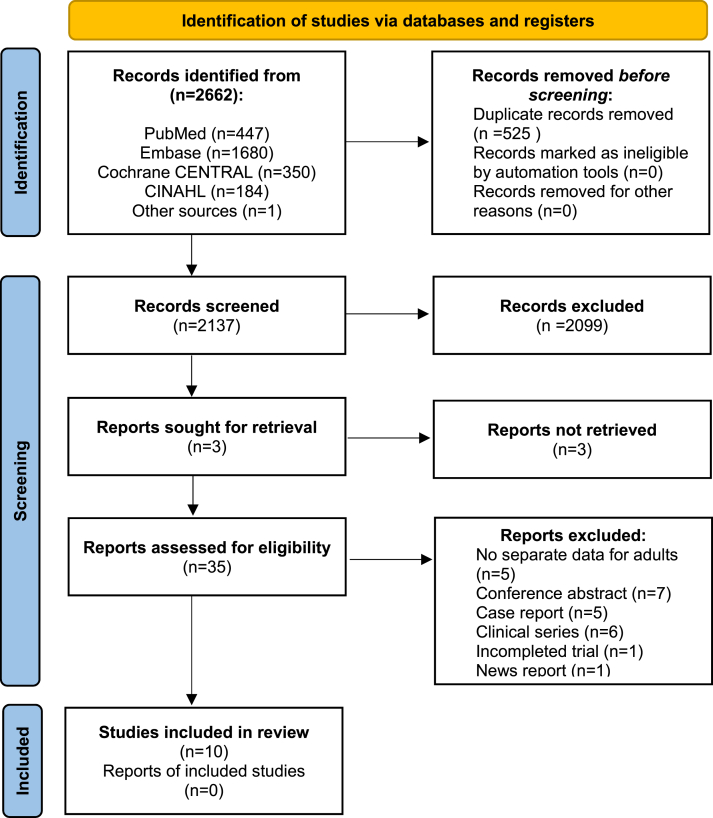
PRISMA flow chart of literature search and article selection.

### 3.2 Study characteristics

**Fig. 2:**
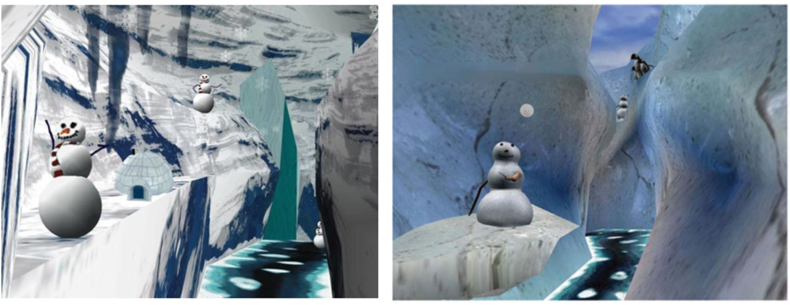
Screenshot of the most widely used software application in the included studies; SnowWorld, designed at the university of Washington [46].

Seven non-crossover RCTs and three crossover RCTs were included with a total of 683 included patients. One of the non-crossover RCTs was a pilot study [44]. Intervention characteristics and measured outcomes are shown in Table 1, Table S1, Table S2 and Fig. 2 [46]. In eight of the 10 studies, VR was investigated during dressing changes, while the remaining studies evaluated the effects of VR during wound debridement [41,45]. Different VR hardware were used in the studies, as shown in Table S1. In all studies, a VR intervention was used as an adjunct to routine wound care. Eight included RCTs were a two-armed trial. Two included RCTs were three-armed trials.

**Table 1:** Characteristics of the included studies.

First author (year)	Country	Study design	Setting	Study population	Type of wound	Type of WC	Participants at baseline (n,%)	Participants at follow-up	Male, %	Mean age, years	Baseline Pain Score, mean ± SD
Guo et al. (2014)	China	Single-centre RCT	An outpatient surgical treatment facility	Patients with hand injuries	Burns: soft tissue defect [5]; cuts [10]; skin avulsion [14]; and nail bed, finger, and hand damage [20].	Dressing changes	Intervention: 49 (50 %) Control: 49 (50 %)	No FU	Intervention: 92 Control: 82	Intervention: 30 Control: 32	Intervention: 6.5 ± 2.2 Control: 6.5 ± 3.1
Ding et al. (2019)	China	Three-centre RCT	Hospitals performing Milligan-Morgan haemorrhoidectomy	Patients after haemorrhoid surgery	Haemorrhoid surgery wounds	Dressing changes: removing the dressings, cleaning and sterilizing the wound, wound assessment and covering the wound with a new dressing.	Intervention: 91 (50 %) Control: 91 (50 %)	No FU	Intervention: 37 Control: 42	Intervention: 46 Control: 45	Intervention: 7.8 ± 1.1 Control: 8.0 ± 1.2
Konstantatos et al. (2008)	Australia	Single- centre RCT	Tertiary burns referral centre	Patients with burns	Burns	Dressing changes	Intervention: 43 (50 %) Control: 43 (50 %)	No FU	*NR*	Intervention: 36 Control: 41	Intervention: 2.7 ± 2.1 Control: 8.0 ± 1.2
Ebrahimi et al. (2018)	Iran	Single-centre RCT	In the burns ward of the hospital	Patients with burns	Burns	Dressing changes	Intervention 1: 20 (33 %) Intervention 2: 20 (33 %) Control: 20 (33 %)	No FU	Intervention 1: 45 Intervention 2: 70 Control: 55	Intervention 1: 32 Intervention 2: 34 Control: 39	Intervention 1: NR Intervention 2: NR Control: NR
Maani et al. (2011)	USA	Crossover RCT	US soldiers burned in combat attacks involving explosive devices	Soldiers with war blast wounds	Blast wounds	Debridement	Intervention: 12 Control: 12	No FU	Intervention: 100 Control: 100	Intervention: 22 Control: 22	Intervention: NR Control: NR
de Araujo et al. (2021)	South America	Single- centre crossover RCT	Stomatherapy clinic	Patients in a stomatherapy clinic with chronic wounds	Neuropathic ulcers, venous ulcers	Dressing changes	Intervention: 17 Control: 17	No FU	Intervention: 88 Control: 88	NR	Intervention: 5.3 ± 2.4 Control: 5.7 ± 2.4
McSherry et al. (2018)	USA	Single- centre crossover RCT	Community-based hospital with American Burn Association-verified regional inpatient burn center	Patients with burns	Burns (83 %), Necrotizing fasciitis or decubitis ulcers (17 %)	Dressing changes	Intervention: 15 Control: 15	No FU	Intervention: 72 Control: 72	Intervention: 38 Control: 38	Intervention: 6.9 ± 2.4 Control: 6.3 ± 2.6
Zheng et al. (2023)	China	Single- centre RCT	Day treatment Centre	Patients with perianal abscess	Perianal abscess	Dressing changes	Intervention: 86 Control: 86	No FU	Intervention: 65 Control: 63	Intervention: 46 Control: 46	Intervention: 4.2 ± 1.2 Control: 4.3 ± 1.3
Armstrong et al. (2023)	USA	Single-centre pilot RCT	Burn Centre	Patients with acute burn injury	Burn injury	Dressing changes	Intervention 1: 4 Intervention 2: 4 Control: 6	2–6 weeks after discharge, about opioid use	Total population: 71	Total population: 38	NR
Park et al. (2023)	USA		Wound clinic	Patients undergoing sharp surgical wound debridement	Venous stasis Diabetic ulcer Breast from surgical complication	Sharp surgical wound debridement	Intervention: 15 Control: 10	No FU	Intervention: 53 Control: 60	NR	NR

Abbreviations: RCT = Randomized Controlled Trial; FU= Follow-up; NR= Not Reported.

### 3.3 Quality of included studies

**Fig. 3:**
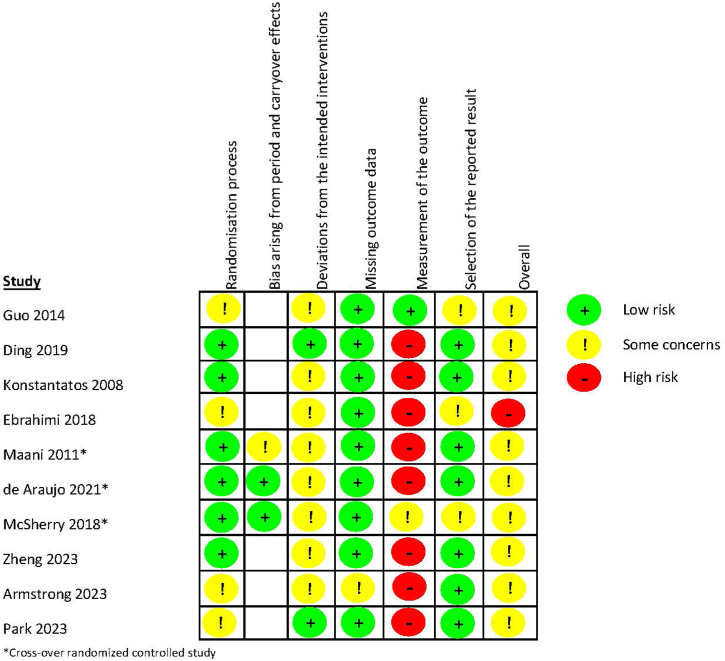
Risk of Bias summary

Fig. 3 shows the summary of the Risk of Bias of the included studies. All studies scored some concerns in most domains, especially in the domain deviations from the intended interventions due to unpublished study protocols. The study of Ebrahimi et al. was the only study that had a high overall risk, and the remaining nine studies had an overall risk with some concerns [28,[38], [39], [40], [41], [42], [43], [44], [45]]. See Appendix S2**.** for a detailed judgement of the quality assessment.

### 3.4 Primary outcome

Figure S1A, Figure S1B, and Table 2 are showing the primary outcome data, expressed as mean pain score during the total wound care procedure [28,[38], [39], [40],^42^,^45^]. Five studies reported a significant difference in pain reduction in the VR group compared to the control group [28,38,39,41,43]. Only Konstantatos et al. reported a significant difference in pain reduction in favour of the control group [40]. Ebrahimi et al. presented only one p-value (p > 0.05) in favour of the multi-media group, without reporting clear results [37]. Two studies reported no significant difference [42,45]. De Araujo et al. also measured the VAS during wound care, but this was reported in n(%), which was also significant [28]. Armstrong et al. reported the lowest mean overall pain in the active VR group (mean VAS score: dressing 1 = 41.3, dressing 2 = 61.0, dressing 3 = 72.7), while those in the passive VR group reported the highest pain (dressing 1 = 58.3, dressing 2 = 74.5, dressing 3 = 89.0) at all three dressing changes, no p-value reported [44]. There was substantial heterogeneity among both non-crossover RCTs (I^2^ = 98 %), and crossover RCTs (I^2^ = 94 %). Given the substantial heterogeneity of the studies, pooling of the data was inappropriate for the primary outcome.

**Table 2:** Primary outcome, mean pain score of all wound care moments.

	Study	Measurement	Intervention				Comparison				Results		
			Mean	SD	Median [IQR]	N	Mean	SD	Median [IQR]	N	MD	*p-*value	Significance
**During wound care**	Konstantatos et al. (2008)	VAS	7.3	NR	NR	43	5.3	NR	NR	43	NR	0.003	Significant^b^
	Ding et al. (2019)	VAS	a	a	a	a	a	a	a	a	a	<0.05	Significant^c^
	Maani et al. (2011)	GRS	4.5	NR	NR	12	6.3	NR	NR	12	NR	<0.05	Significant^c^
	Zheng et al. (2023)	VAS	a	a	a	a	a	a	a	a	a	<0.05	Significant^c^
	Park et al. (2023)	Likert scale	2.5	NR	NR	15	2.2	NR	NR	10	NR	NR	Not significant
	Armstrong et al. (2023)^e^	VAS	NR	NR	NR		NR	NR	NR		NR	NR	NR
	Ebrahimi et al. (2018)	VAS	～	～	～	～	～	～	～	～	～	<0.05	Significant^d^
**After wound care**	Guo et al. (2014)	VAS	2.6	3.1	NR	49	7.6	3.4	NR	49	−5.00 [-6.03, −3.99]	0.000	Significant^c^
	Ding et al. (2019)	VAS	4.3	1.3	NR	91	4.3	1.3	NR	91	−0.0 [-0.40-0.36]	NR	Not significant
	Konstantatos et al. (2008)	VAS	3.7	NR	NR	43	2.3	NR	NR	43	NR	0.031	Significant^b^
	de Araujo et al. (2021)	VAS	1.0	1.6	NR	17	6	2.4	NR	17	−5.00 [-6.39,-3.61]	<0.001	Significant^c^
	McSherry et al. (2018)	VAS	5.8	2.9	NR	15	5.7	2.6	NR	15	0.1 [-1.87, 2.07]	>0.05	Not significant
	Zheng et al. (2023)	VAS	5.3	1.2	NR	86	5.2	1.6	NR	86	NR	>0.05	Not significant
	Park et al. (2023)	Likert scale	2.5	NR	NR	15	2.3	NR	NR	10	NR	NR	Not significant

Abbreviations: VAS= Visual Analogue Scale; GRS = Graphic Rating Scale; NR = not reported.

～Ebrahimi et al. reported no clear results.

a Pain scores at 5, 10, 15 and 20 min during first dressing change with or without Virtual Reality. No absolute data, p < 0.05 for all comparisons.

b Significant in favour of the non-Virtual Reality group.

c Significant in favour of Virtual Reality.

d Significant in favour of the multi-media group.

e Armstrong et al. reported the mean of 3 wound care procedures separately.

### 3.5 Secondary outcomes

Seven studies reported one of the predefined secondary outcomes [28,[39], [40], [41]]. The secondary outcomes were expressed as (mean ± SD). McSherry et al. and Park et al. found no significant differences in anxiety scores between the groups (*p* > 0.05) [42]. Guo et al. reported only baseline anxiety [39].

Pain medication use was reported in three studies [40,42,44]. Konstantatos et al. reported no significant differences in the amount of intravenous opioids used during the procedure in the intervention group (VR + PCA group) (21.6 ± 17.3) and the control group (PCA group) (25.0 ± 18.5) (*p* = 0.4) [40], while McSherry et al. reported a significant difference before and during dressing changes (VR 91.7 ± 10.1; CG 103.1 ± 16.1, *p* = 0.02) [42]. In contrast, Armstrong et al. observed less use of pain medication, morphine, in the control group compared with the active VR and passive VR group during all 3 wound care moments [44]. However, no p-value was reported. Three studies reported vital signs [28,38,43]. De Araujo et al. measured SBP (VR 137 ± 16.2; CG 131 ± 12.9, *p* = 0.012), DBP (VR 86 ± 8.1; CG 90 ± 3.2, *p* = 0.004), heart rate (VR 79 ± 4.9; CG 89 ± 5.7, *p* = 0.001) and blood oxygen level (VR 98 ± 1.6; CG 98 ± 1.6, *p* = 0.317) [28]. Ding et al. reported no significant differences in SaO_2_ and pulse rate between the VR and control group during the dressing changes, but data is not published [38]. Zheng et al. observed no difference in pulse rate between the groups during dressing changes (*p* > 0.05) and stable and normal SaO_2_ levels (no *p*-value reported) [43]. Three studies reported patient satisfaction [28,42,44]. De Araujo et al. showed that 64.7 % (n = 11) of the participants were extremely satisfied, and 94.1 % (n = 16) reported no discomfort [28]. McSherry et al. reported that >75 % of participants found VR therapy helpful and made the connection for them [42]. Armstrong et al. reported no patient satisfaction results [44]. No study reported about provider satisfaction.

## 4 Discussion

This systematic review of the current literature on use of VR to mitigate pain scores during wound care reveals conflicting evidence, which causes the included studies to be inconclusive. Five studies indicate a significant reduction in pain during or immediately after wound care that can be attributed to the of use of VR, while three other studies fail to show any effect. Unfortunately, due to the substantial heterogeneity between studies, conducting a meta-analyses on these results must be considered statistically inappropriate. Use of intravenous opioids and effects on vital signs were reported in only a few studies, again with conflicting results. And from our review, no evidence-based statements about changes in vital signs between the groups with VR and without VR can be stated. Patient satisfaction was reported in only two studies, both showing patient satisfaction with the possibility of using on-demand VR during wound care. Looking into literature, another systematic review of 26 articles was able to report favourable about the significance of VR in reducing in pain and anxiety rates in patients. However, this systematic review included very heterogenic studies. VR was used as a distraction across various settings (e.g. dental care, radiography) in populations ranging from young children to adults. It is hence difficult to conclude any effect for specific wound care. The same conclusion can be drawn from the overview study of Dreesman et al., which included 23 articles [22]. In our systematic review, we focused only on evaluating the effectiveness of VR within the context of wound care, without distinguishing between different types of wound care and methods. As a result, we were able to include 10 articles.

The difference in results found in the included studies in our review may perhaps be contributed to the differences in visual scenery as provided by the VR systems. Patient preference, which is important for shared decision-making and for its impact on the treatment outcome itself, was often not considered in the included studies [[37], [38], [39], [40], [41], [42],^44^]. This is surprising, as studies indicate that effectiveness may depend greatly on tailoring interventions to patient preferences. [[47], [48], [49], [50]] In the context of VR glasses, limited patient choice could lead to reduced distraction and potentially compromise effectiveness. The effectiveness of any VR intervention is also influenced by the experienced level of immersion [51,52]. From our review, we were unable to retrieve if immersiveness levels were reported by patients. Yet another possible factor influencing the effect of VR is timing. It matters when VR glasses are used during treatment [53]. If too late, one may not be immersed enough to be distracted. In all included studies in this systematic review, VR glasses were used during wound care. But none of the information provided indicates the time window between receiving VR treatment and initiating wound treatment. Finally, we found that some patients may find wearing VR glasses uncomfortable, especially when they cannot perceive their surroundings while receiving care [54]. This was neither recorded nor retrievable from studies.

### 4.1 Strengths& limitations

This review has some strength and limitations. One notable strength is the utilization of an extensive and meticulous literature search. Each stage of the review involved the participation of two or three independent reviewers, ensuring reliability. Additionally, established tools were employed for quality assessments. In the current study, we conducted a broad search, which included video glasses, augmented reality, intra- and extramural setting. Furthermore, we used validated scales for primary outcome assessment. By doing so, we were able to draw some conclusions on what is missing in current research, as outlined in the discussion. The main limitation lies in our inability to define the minimum important difference (MID) in pain. Given the large variation in study design and sample size between studies, calculating the MID was not feasible [55,56]. Therefore, we cannot comment on the smallest change in pain outcome measure that patients or researchers consider clinically relevant. The included studies did not all provide a clear description of wound care and VR intervention. Moreover, a subgroup analysis could not be conducted to understand if 1)individual preference vs. no individual preference; 2)timing of VR; and 3)level of distraction played a role in the effect of the VR intervention. We could only analyze nine articles, no clear data had been abstracted from one study [37]. Despite our best efforts, we were unable to contact several authors to obtain data from papers that could have potentially contributed to this research. Furthermore, in our search, we compared VR with usual care. But usual care to date also means that many patients usually use a tablet or phone to distract themselves, during their wound care. Therefore, instead of excluding this group, we included those who received an additional intervention in addition to standard care in our study.

### 4.2 Future perspectives

The attitude of healthcare professionals towards VR implementation is crucial, as it can significantly influence acceptance and effectiveness. Positive attitudes among healthcare professionals can promote the integration of VR into clinical practice, while negative perceptions can hinder its adoption [14,57]. The lack of established guidelines for the use of VR in healthcare settings contributes to this challenge. Without clear guidelines, there is considerable variation in the use of VR in different studies and clinical settings. Research is needed to determine optimal protocols, including the timing and duration of VR use before procedures and their frequency, including larger study population, with a choice of several VR videos, multiple outcome measures such as anxiety, opioid use, vital signs, and patient- and provider satisfaction. Currently, there is no guideline recommending the optimal use of VR for pain and anxiety reduction. This should be available in the near future.

### 4.3 Conclusion

Our systematic review shows a lack of robust methodology and perhaps as a result, conflicting findings regarding the effectiveness of VR as an effective non-pharmacological distraction method for pain in adult patients undergoing wound care are inconclusive. Evidence is too limited to draw any conclusions about the secondary outcome measures as well. This systematic review highlight the issues resulting and the need for more high-quality RCTs to truly state the impact of using VR in wound care. Future studies should include a large study population, measure more outcome measures, explore the best time to use VR, and offer multiple scenarios.

## CRediT authorship contribution statement

**H. Demirci:** Writing – review & editing, Writing – original draft, Visualization, Project administration, Methodology, Data curation, Conceptualization. **T. Lachkar:** Writing – original draft, Data curation. **W.X.I. Fleur:** Writing – original draft, Methodology, Data curation. **E.Z. Barsom:** Writing – review & editing, Methodology, Conceptualization. **A.M. Eskes:** Writing – review & editing, Methodology. **M.P. Schijven:** Writing – review & editing, Supervision.

## Declaration of competing interest

The authors declare that they have no known competing financial interests or personal relationships that could have appeared to influence the work reported in this paper.

## Figures and Tables

**figs1:**
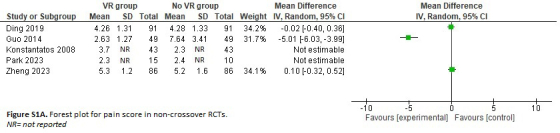


**figs2:**