# Supplementary material for: The effect of virtual reality therapy on pain and anxiety during wound care in adults: A systematic review
Source: Heliyon. 2024 Dec 7;10(24):e40858. doi: 10.1016/j.heliyon.2024.e40858 (PMC11681856; doi:10.1016/j.heliyon.2024.e40858)
Supplement: Multimedia component 1 [file mmc1.docx]

|  |  |  |  |  |  |  |  |  |  |  |  |
| --- | --- | --- | --- | --- | --- | --- | --- | --- | --- | --- | --- |

| **Table S1.** Description of interventions, comparisons, and outcomes. | | | | | | | | | | | |
| --- | --- | --- | --- | --- | --- | --- | --- | --- | --- | --- | --- |
| **First author (year)** | **Intervention** | **Comparison** | **Primary outcome** | **Secondary outcomes** | | | | | | | |
|  | | | |  |  |  |  |  |  |  |  |
|  |  |  | Pain | Anxiety | SBP | DBP | HR | SaO_2_ | Opioid | Patient Satisfaction |  |
| Guo et al. (2014) | Intervention:  *VR*  Type of hardware: *VR + headphones*  Type of content:  *3D film ‘Afanda’*  Duration VR:  *Patients were asked to watch 3D movies for 5 minutes before the dressing change ended*  Duration WC:  *NR*  Duration VR+ WC:  *NR* | Control:  *No VR equipment. Patients were asked to close their eyes and accept a conventional dressing repose until dressing was completed*  Duration of WC/CG:  *NR* | V | *Only baseline* |  |  |  |  |  |  |  |
| Ding et al. (2019) | Intervention:  *VR*  Type of hardware: *eMagin Z800 3DVISOR*  Type of content:  *SnowWorld*  Duration VR:  *NR*  Duration WC:  *NR*  Duration VR+ WC:  *21 min* | Control:  *received the standard dressing change procedure*  Duration of WC/CG:  *20 min* | V |  |  |  | V; *Data not shown* | V;  *Data not shown* |  |  |  |
| Konstantatos et al. (2008) | Intervention:  VR+PCA  Type of hardware:  *VR goggles*  Type of content:  *SnowWorld*  Duration VR: *NR*  Duration WC:  *NR*  Duration VR+WC:  *77 min* | Control:  PCA  Duration WC/CG:  *77 min* | V |  |  |  |  |  | V |  |  |
| Ebrahimi et al. (2018) | Intervention 1:  VR  Type of hardware:  *VR-specific headset*  Type of content:  *Images and waterfall sounds*  Duration VR:  *NR*  Duration WC:  *NR*  Duration VR+WC:  *NR* | A. Intervention 2:  Multimedia  Type of hardware:  *LCD*  Type of content:  *Images and waterfall sounds*  Duration MM:  *NR*  B. Control:  *Not described*  Duration CG:  *NR* | V: *results not clearly reported* |  |  |  |  |  |  |  |  |
| Maani et al. (2011) | Intervention:  *Standard analgesic medications+ VR*  Type of hardware:  *SR-80A VR goggles*  Type of content:  *SnowWorld*  Duration VR:  *6 min*  Duration WC:  *6 min*  Duration VR+WC:  *6 min* | Control:  *Only standard analgesic medications* | V |  |  |  |  |  |  |  |  |
| de Araujo et al. (2021) | Intervention:  *VR*  Type of hardware:  *Oculus Go®*  Type of content:  *360^0^ video images and location-specific spatial sounds*  Duration VR:  *22 min*  Duration WC:  *22 min*  Duration VR+WC:  *22 min* | Control:  No VR  Duration WC:  *22 min* | V |  | V | V | V | V | V | V |  |
| McSherry et al. (2018) | Intervention:  *VR + IV Opioid*  Type of hardware:  *VR goggles (NVISING MX 90, NVISING, reston VA), earphones*  Type of content:  *SnowWorld*  Duration VR:  *NR*  Duration WC:  *Nr*  Duration VR+WC:  30 min | Control:  *Only IV Opioid*  Duration of WC+CG:  *30 min* | V | V |  |  |  |  | V | V |  |
| Zheng et al. (2023) | Intervention: VR Type of Hardware: Pico G2 4K head-mounted display  with a 101° view field, and a hand-held controller. Type of content: Choice of immersive 360°  Cine-VR scene of movies  Duration VR: *NR*  Duration WC: *NR* Duration VR + WC: 23 min | Control: No VR. Received the standard daily dressing changes. Duration of WC/CG:  23 min | V |  |  |  | V | V |  |  |  |
| Armstrong et al. (2023) | Intervention 1:  Active VR  Type of hardware 1+2: Low-cost VR headset+ an Apple iPhone XS and Bluetooth gaming controller  Type of content 1: Four VR games; projectile (town and cave) and rhythm game (city and forest) Duration WC+VR 1: 1^st^ WC 49.5 min 2^nd^ WC 44 min 3^rd^ WC 51 min Intervention 2:  Passive VR  Type of content 2: Automated virtual tour of the same virtual environments as type of content 1, without interactive elements of the games.  Duration WC+VR 2: 1^st^ WC 52.5 min 2^nd^ WC 56.5 min 3^rd^ WC 64 min | Control: Received the routinely used distraction tools (i.e. music and/or talking) Duration of WC/CG: 1^st^ WC 59 min  2^nd^ WC 60 min  3^rd^ WC 49 min | V |  |  |  |  |  | V | V;  *Data not shown* |  |
| Park et al. (2023) | Intervention: VR  Type of hardware: Samsung Galaxy S7 and a Samsung Gear Virtual  Reality headset with headphones  Type of content: A playlist chosen by the research team of freely available 360 degree field of view media from YouTube.  Duration of VR+WC:  5.1 min | Control:  No VR  Duration of WC/CG: 7.4 min | V | V |  |  |  |  |  |  |  |
| *Abbreviations: VR= Virtual Reality; WC= Wound Care; CG= Control Group; min= minutes; HR= Heart Rate; SatO_2_= Oxygen blood levels; NR= Not Reported* | | | | | | | | | | | |
