# Supplementary material for: The effect of virtual reality therapy on pain and anxiety during wound care in adults: A systematic review
Source: Heliyon. 2024 Dec 7;10(24):e40858. doi: 10.1016/j.heliyon.2024.e40858 (PMC11681856; doi:10.1016/j.heliyon.2024.e40858)
Supplement: Multimedia component 2 [file mmc2.docx]

|  |  |  |  |  |  |  |  |  |  |  |  |
| --- | --- | --- | --- | --- | --- | --- | --- | --- | --- | --- | --- |

| **Table S2.** Description of the primary outcome measurement | | | | | |
| --- | --- | --- | --- | --- | --- |
| **First author (year)** | **Measurement** | **Time points** | **Time start intervention** | **Number of WC procedures** | **Time of WC procedures** |
| Guo et al. (2014) | Visual Analog Scale (VAS), 0-10 | Within 5 min of the dressing change: three times at two  time points and were used to calculate the average values | 5 min before the dressing  change ended | 3 | *NR* |
| Ding et al. (2019) | Visual Analog Scale (VAS), 0-10 | Before dressing change, 5-min intervals until the completion of the dressing change, 5 min after the completion of the dressing change | NR *(Dressing changes proceeded after the patient*  *fully immersed in the VR distraction*) | 1 | The first dressing change after surgery (postoperative day 2) |
| Konstantatos et al. (2008) | Visual Analog Scale (VAS), 0-10 | During- and after dressing change | Prior to dressing change | 5 | *NR* |
| Ebrahimi et al. (2018) | Visual Analog Scale (VAS), 0-10 | Once on a wound care day, before intervention | *NR* | 5 | Five consecutive days from the third day to the seventh day  of burns |
| Maani et al. (2011) | Graphic Rating Scale (GRS), 0-10 | During two brief pauses in the wound care procedure (once after each 6 minute wound care period), each patient completed three subjective pain ratings using GRS labeled 0 to 10 with respect to the preceding 6 minutes of wound care | The 12-min segment was divided into two equivalent wound care segments (∼6 minutes per segment). Patients received VR first or second. | 1 | After painful wound care |
| de Araujo et al. (2021) | Visual Analog Scale (VAS), 0-10 | Two minutes before starting dressing application, after debridement, immediately after completion of the whole procedure. And worst/mildest pain score in the last 24 hours. Time between two observations was seven days. | The time for changing dressings was chosen for the use of VR. | 2 | Time between two observations was seven days. |
| McSherry et al. (2018) | Verbal Numeric Scale (VNS), 0-10 | Before- and after dressing change | After preprocedure medication  administration, a few minutes before the wound care | 2 |  |
| Zheng et al. (2023) | Visual Analog Scale (VAS), 0-10 | All outcome measurements were performed before the  dressing change and then at 5 min intervals until end of  the dressing change by the same nurse in our nursing  team. At 5 min after the end of the dressing changes,  outcome measurements were recorded as the post-dressing  change outcome. | Dressing changes were performed following  fully immersing in VR distraction | 1 | First dressing change after  surgery |
| Armstrong et al. (2023) | Visual Analog Scale (VAS), 0-100 | Immediately following the dressing change. This process was completed once per day for up to three inpatient dressing changes. | During. However, is not clearly described. | 3 | *NR* |
| Park et al. (2023) | Likert scale, 0-10 | Pre-debridement, peri-debridement, and immediately postdebridement | The virtual reality headsets were  given to the patients to wear while the patients had their  wounds cleaned and prepared with topical lidocaine, by  the nursing staff before the procedure. | 1 | During sharp surgical wound debridement |
| *Abbreviations: min= minutes; NR= Not Reported* | | | | | |
